# Simultaneous Electrochemical Exfoliation of Graphite and Formation of Azide-Functionalized Graphene Flakes and Their Application in Sensing

**DOI:** 10.1021/acsomega.6c01334

**Published:** 2026-04-18

**Authors:** Selene Muñoz-Vargas, Marcos Fernando Perez-Pucheta, Karl S. Coleman

**Affiliations:** † Department of Chemistry, Durham University, South Road, Durham DH1 3LE, U.K.; ‡ Department of Chemistry, School of Physical Sciences, 4591 University of Liverpool, Peach Street, Liverpool L69 7ZE, U.K.

## Abstract

Electrochemical exfoliation has emerged as a facile method for graphene production. This work presents a one-step electrochemical synthesis of bulk azidated graphene by exfoliating graphite foil in a sodium sulfate/sodium azide aqueous solution. This approach circumvents the limitations associated with traditional methods that utilize graphene oxide (GO) as a precursor, which often require subsequent reduction steps that can compromise the electrical conductivity and potentially convert azide groups to amines. Here, we directly introduced azide functionalities onto the graphene surface during the exfoliation process, yielding high quantities of conductive few-layer graphene. To demonstrate the utility of this azidated graphene, we successfully performed copper­(I)-catalyzed alkyne–azide cycloaddition (CuAAC) click chemistry to attach a thiourea-based selector molecule for the selective gas detection of cyclohexanone, a nonexplosive vapor marker. The resulting sensor exhibited a selective response toward cyclohexanone with a low limit of detection of 4.5 ppm. The enhanced affinity for cyclohexanone was attributed to its higher polarizability compared with other volatile organic compounds. This work highlights the advantages of a simple and cost-effective electrochemical route for producing functionalized graphene using click chemistry with preserved electrical conductivity, opening up possibilities for a range of applications that would benefit from a tailored functional surface.

## Graphical abstract

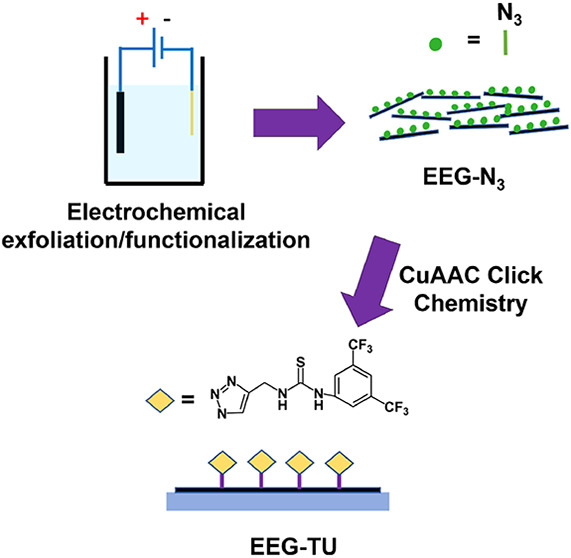

## 1 Introduction

In recent years, electrochemical exfoliation of graphite has emerged as a simple method to produce graphene materials. It is an attractive strategy to produce few-layer graphene because it is economical, environmentally friendly, and not equipment-intensive and operates at ambient temperature and pressure conditions.^1^ Most importantly, electrochemical exfoliation can readily produce a mixture of single and few-layer graphene, with the procedure completed quickly and able to produce gram-scale quantities of flakes with yields as high as 80%.^2–4^ Furthermore, the sheet size, carbon/oxygen (C/O) ratio, solubility, and electrical conductivity can be modified by controlling process parameters, such as applied electrical potential, current, processing time, and the composition of electrolytes.^5^

A typical setup includes a working electrode, counter electrode, electrolyte, and power supply. An applied electrical potential can drive positively or negatively charged ions from the electrolyte into the graphitic interlayers.^6^ It has been shown that the structural imperfections in graphite foils facilitate the exfoliation of graphite and can reduce damage caused by oxidative reactions during the electrochemical exfoliation.^7^

Previous work on the exfoliation and azidation of highly oriented pyrolytic graphite flakes indicates that graphene could be simultaneously exfoliated and functionalized in large quantities by employing larger substrates, including graphite foil.^8^ From the many functional groups that could be introduced to the graphene surface, azide groups offer the most potential. They can be used to react with an alkyne-terminated molecule, using the copper­(I)-catalyzed alkyne–azide cycloaddition (CuAAC) click chemistry, paving the way to a plethora of other functional groups being introduced to the graphene surface.^9^ The versatility of this functional group has found application in the development of sensors for copper detection,^10^ biosensing,^11^ and ultrafiltration technology,^12^ among others.

The preservation of graphene’s electrical conductivity during azide group functionalization is a key objective in sensor development. This poses a challenge as in most methods, graphene oxide is used as a precursor for covalent^10–17^ or noncovalent functionalization,^18^ and a further reduction step of the graphene material is necessary to improve conductivity, which can lead to the conversion of azide groups to amino groups.^19^ Therefore, it is essential to find new ways to introduce azide groups to the graphitic surface, while keeping the conjugated lattice predominately intact. In previous work by Ustavytska et al.,^20^ graphene was exfoliated for 20 h in a 1 M sodium azide solution in the absence of any other intercalating agent. Their investigation demonstrated that nitrogen was introduced in some way to the graphitic lattice but did not show what functional groups were introduced. Whereas, recent research by Li et al. demonstrated that simultaneous electrochemical exfoliation in a sodium sulfate (Na_2_SO_4_)/sodium azide (NaN_3_) solution can introduce azide groups to graphene flakes. However, this process was limited to the exfoliation of a single flake of graphite or highly oriented pyrolytic graphite.^8,21,22^

Motivated by this, we investigated the bulk exfoliation of graphite foil in a solution of Na_2_SO_4_/NaN_3_ to produce graphene flakes with azide groups on their surface. Furthermore, we demonstrate that the flakes retain their electrical conductivity even after being functionalized with an alkyne-terminated molecule through CuAAC click chemistry. As a proof of concept, we introduce the 1-(3,5-bis­(trifluoromethyl)­phenyl)-3-(prop-2-yn-1-yl) thiourea molecule to the surface of graphene to aid the selective detection of cyclohexanone, which is a nonexplosive vapor marker used to recrystallize cyclotrimethylenetrinitramine (RDX) in explosive formulations.^23^ It has previously been demonstrated that single-walled carbon nanotubes (SWCNTs) functionalized with thiourea derivatives, either noncovalently or covalently, exhibit a strong response to cyclohexanone.^23–25^ Although a similar molecule was proved to work using carbon nanotubes,^24,25^ here, we show the use of azidated graphene for gas sensors.

## 2 Materials and Methods

### 2.1 Graphene Preparation

Azidated graphene flakes were produced by electrochemical exfoliation of graphite foil by applying a potential of 7 V in 0.2 M Na_2_SO_4_/0.1 M NaN_3_ electrolyte solution using a platinum wire as a counter electrode. A detailed procedure is provided in S1 in the Supporting Information. The selector 1-(3,5-bis­(trifluoromethyl)­phenyl)-3-(prop-2-yn-1-yl) thiourea was synthesized according to the procedure reported in S2 in the Supporting Information. This sensing molecule was attached to the graphene flakes using the copper­(I)-catalyzed alkyne–azide cycloaddition (CuAAC) click chemistry protocol as described in S3 in the Supporting Information.

### 2.2 Material Characterization

The morphology and structure of the electrochemically exfoliated graphene flakes were investigated by SEM (Zeiss Sigma 360 VP microscope at 5.0 kV with an in-lens detector), AFM (SmartSPM-1000 in noncontact mode), TEM (JEOL 2100F FEG microscope operating at 200 kV), and X-ray diffraction (Bruker D8 Venture diffractometer Cu Kα1 source (λ = 1.5406 Å) operating in Braggs β mode). Raman spectra were collected using a Horiba LabRam Evolution confocal Raman microscope with a 50x long-working distance objective (NA = 0.5) and a 532 nm laser operating at ∼1.1 mW. The instrument was calibrated using the Si line at 520.7 cm^–1^. TGA was carried out using a PerkinElmer Pyris I. Samples were heated under a helium atmosphere in a ceramic pan from room temperature to 800 °C at a rate of 10 °C/min. A Kratos Axis Ultra DLD system was used to collect XPS spectra using a monochromatic Al Kα X-ray source operating at 144 W (12 mA × 12 kV). Data were collected with pass energies of 80 eV for survey spectra and 20 eV for the high-resolution scans with step sizes of 1 and 0.1 eV, respectively. The sheet resistance was estimated using the van der Pauw method with a four-point probe and a spacing of 5 mm, using a Keithley 2602 source meter unit (SMU); three different positions were measured.

### 2.3 Device Preparation

Glass slides were cleaned by ultrasonication in acetone for 15 min and dried before use. Using a homemade tape mask, with a 1 mm gap between the metal electrodes, a layer of gold/palladium of 100 nm thickness was deposited onto the glass slide using a sputter coater. The electrodes were placed on a hot plate at 77 °C; then EEG (electrochemically exfoliated graphene) or EEG-TU (electrochemically exfoliated graphene functionalized with a thiourea selector) solution was dropcast between the electrodes until 15–20 kΩ resistance is achieved. They were left on the hot plate at 77 °C for 30 min before recording sensing measurements (Figure S5a, Supporting Information).

### 2.4 Experimental Setup for Testing Graphene-Based Sensors

A gas sensor measurement system was developed by using a vapor-generating bubbler. Concentrations were estimated from the partial pressure of the volatile organic compounds (VOCs) calculated from Antoine’s equation as reported by Kim et al.^26^ It is explained in detail in S4 in Supporting Information. For testing, the devices were placed in a PTFE gas testing chamber, 1 V was applied using a potentiostat (PalmSens4), and the current passing through was recorded using PSTrace 5.9 software provided by Palm Instruments.

The sensors were exposed to various VOCs three times, repeated in triplicate. In every experiment, the exposure time to the analyte and flushing time with the carrier gas (argon) were fixed to 1 min. The normalized response of the sensors was calculated as

1 $$-\frac{\Delta G}{G_{0}}\left(\%\right)=-\frac{I-I_{0}}{I_{0}}\times 100$$

where *I*_0_ is the initial current before exposure to the analyte and *I* is the measured current over time. In this work, the response time was considered as the time interval over which the sensor response is 90% of the final value when it is exposed to a specific gas concentration. The limit of detection (LOD) was considered as the value when the signal-to-noise ratio is equal to 3. More detailed information can be found in S5 in the Supporting Information.

## 3 Results and Discussion

Here, we electrochemically exfoliate graphite foil in 0.2 M Na_2_SO_4_/ 0.1 M NaN_3_ aqueous solution to enable the exfoliation and the introduction of azide groups on the surface of the graphene. This step is followed by the CuAAC click chemistry reaction to introduce the 1-(3,5-bis­(trifluoromethyl)­phenyl)-3-(prop-2-yn-1-yl) thiourea molecule (Figure 1).

**Figure 1:**
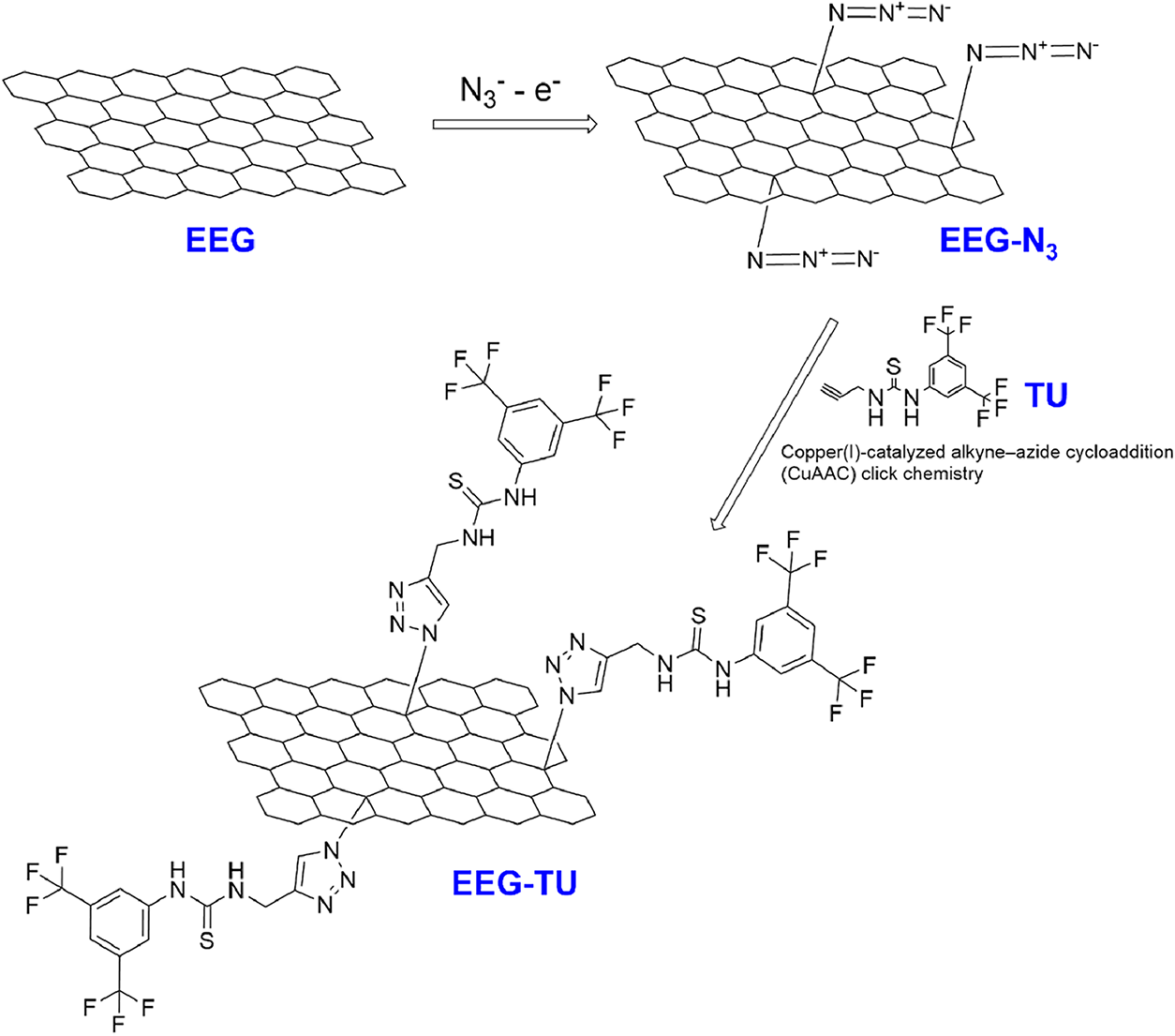
Reaction steps for the attachment of the 1-(3,5-bis­(trifluoromethyl)­phenyl)-3-(prop-2-yn-1-yl) thiourea molecule on the surface of graphene, electrochemically exfoliated/azidated, by CuAAC click chemistry.

The azidated electrochemically exfoliated graphene (EEG-N_3_) and control, electrochemically exfoliated graphene (EEG), were characterized using X-ray powder diffraction analysis to determine the structure of exfoliated graphene sheets, Figure 2. The diffraction peak (002) of EEG and EEG-N_3_ appears at 2θ = 26.47° and 26.58°, respectively, with a corresponding interlayer distance (*d*) of 0.337 and 0.335 nm. In contrast, the (002) peak of the graphite foil appears at 26.86° with a *d*-spacing of 0.332 nm. Compared to graphite, both EEG and EEG-N_3_ showed a slightly lower 2θ angle and increased *d*-spacing, due to the introduction of sulfate ions, oxygen, and azide groups from electrochemical exfoliation. The (002) peaks were also notably broader, indicative of the exfoliation of graphite layers and reduction of the crystallite size.^27,28^

**Figure 2:**
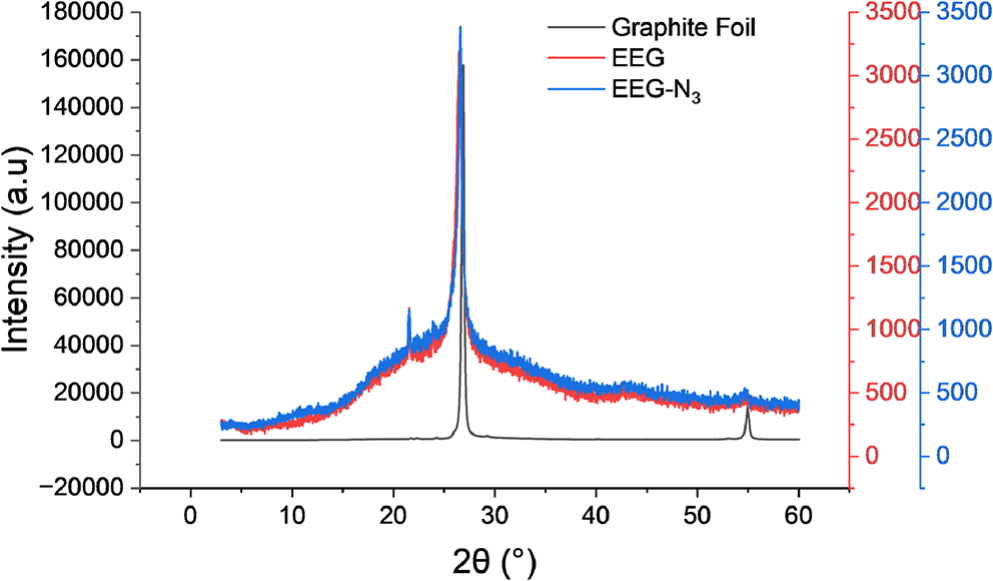
X-ray diffraction (XRD) pattern of the graphite foil (black), EEG (red), and EEG-N_3_ (blue).

The scanning electron microscopy (SEM) images of the surface and edge morphologies of the graphite foil after exfoliation at 7 V in Na_2_SO_4_ and Na_2_SO_4_/NaN_3_ aqueous solutions are shown in Figure 3a,b, respectively. A network of wrinkles on the surface of the graphite can be identified, which is attributed to the expansion and swelling of the graphite layers caused by gas evolution, which is visible during the exfoliation process. According to Parvez et al., during the electrochemical process, the edge and grain boundaries of the graphite electrode open up first, which facilitates anion intercalation and results in exfoliated graphene sheets,^1^ as observed in the images.

**Figure 3:**
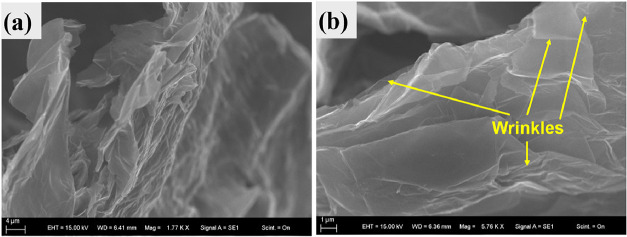
SEM images of the graphite foil electrochemically exfoliated at 7 V in (a) 0.4 M Na_2_SO_4_ and (b) exfoliated in 0.2 M Na_2_SO_4_/ 0.1 M NaN_3_ aqueous solution, where the expanded graphite layer and wrinkles are observed.

After sonication, the samples were deposited on Si/SiO_2_ and the size and thickness distributions were measured using AFM (Figure 4a–d). The lateral size distribution from 80 flakes measured (Figure 4c) revealed that over 90% of the EEG-N_3_ sheets are larger than 1 μm, with the largest flake size observed being ∼9.5 μm. The thickness distribution (Figure 4d) indicates that 90% of the EEG-N_3_ sheets are few-layer graphene (thickness <3.5 nm). The presence of monolayer and few-layer graphene following exfoliation was confirmed using TEM. Figure 5a,b shows two suspended EGG-N_3_ sheets, and their respective selected area electron diffraction (SAED) patterns (Figure 5c,d), and the corresponding intensity profiles (Figure 5e,f). According to Meyer et al., monolayer graphene film shows a stronger diffraction intensity for the (01̅10) plane than the (12̅10) plane, with the opposite true for bilayer or trilayer graphene films.^29,30^ Thus, the profile shown in Figure 5e corresponds to multilayer graphene and the profile in Figure 5f corresponds to a monolayer graphene sheet.

**Figure 4:**
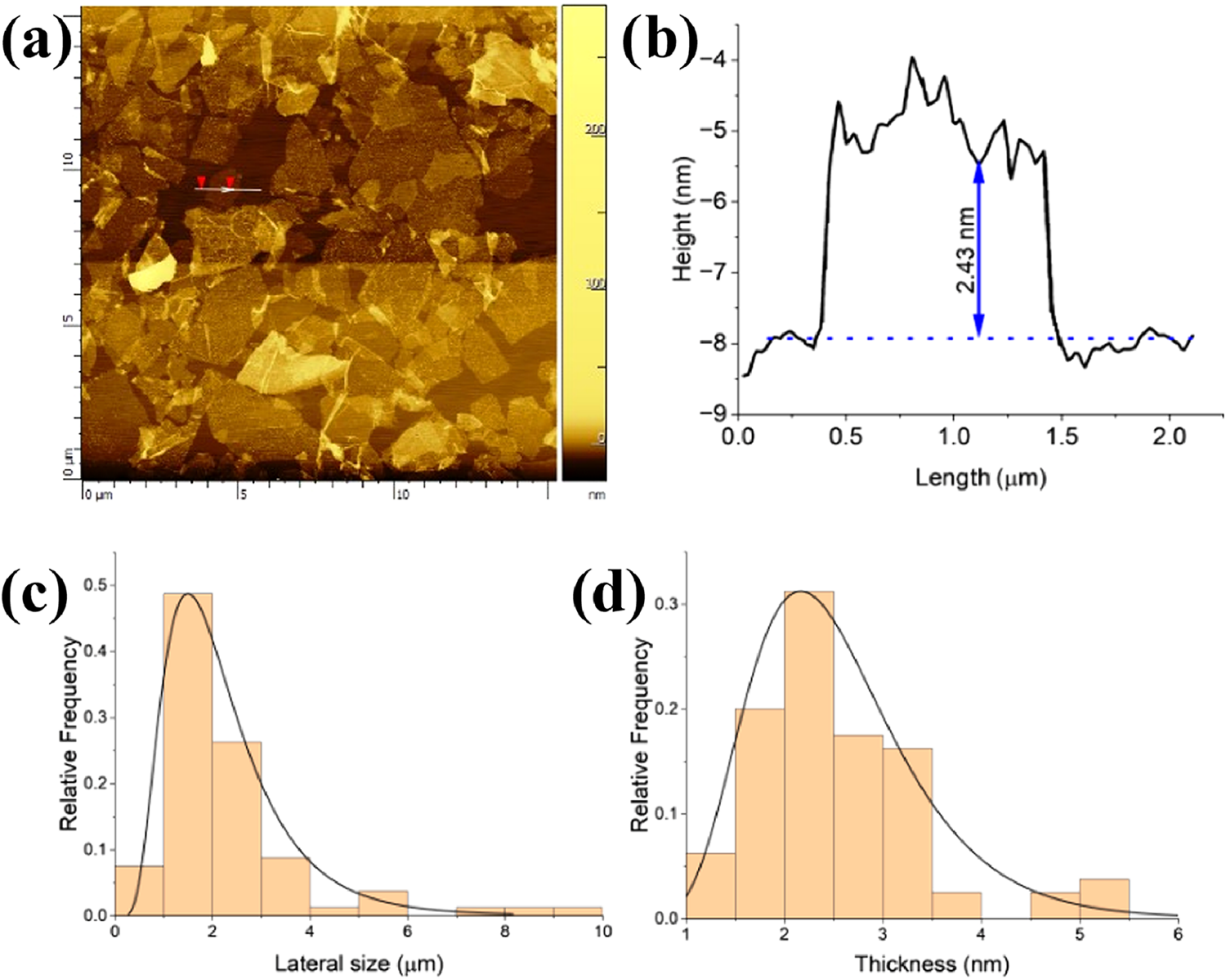
(a) AFM image of EEG-N_3_ and (b) typical height profile of a flake from (a). (c, d) Lateral size and thickness distributions taken from 80 individual flakes.

**Figure 5:**
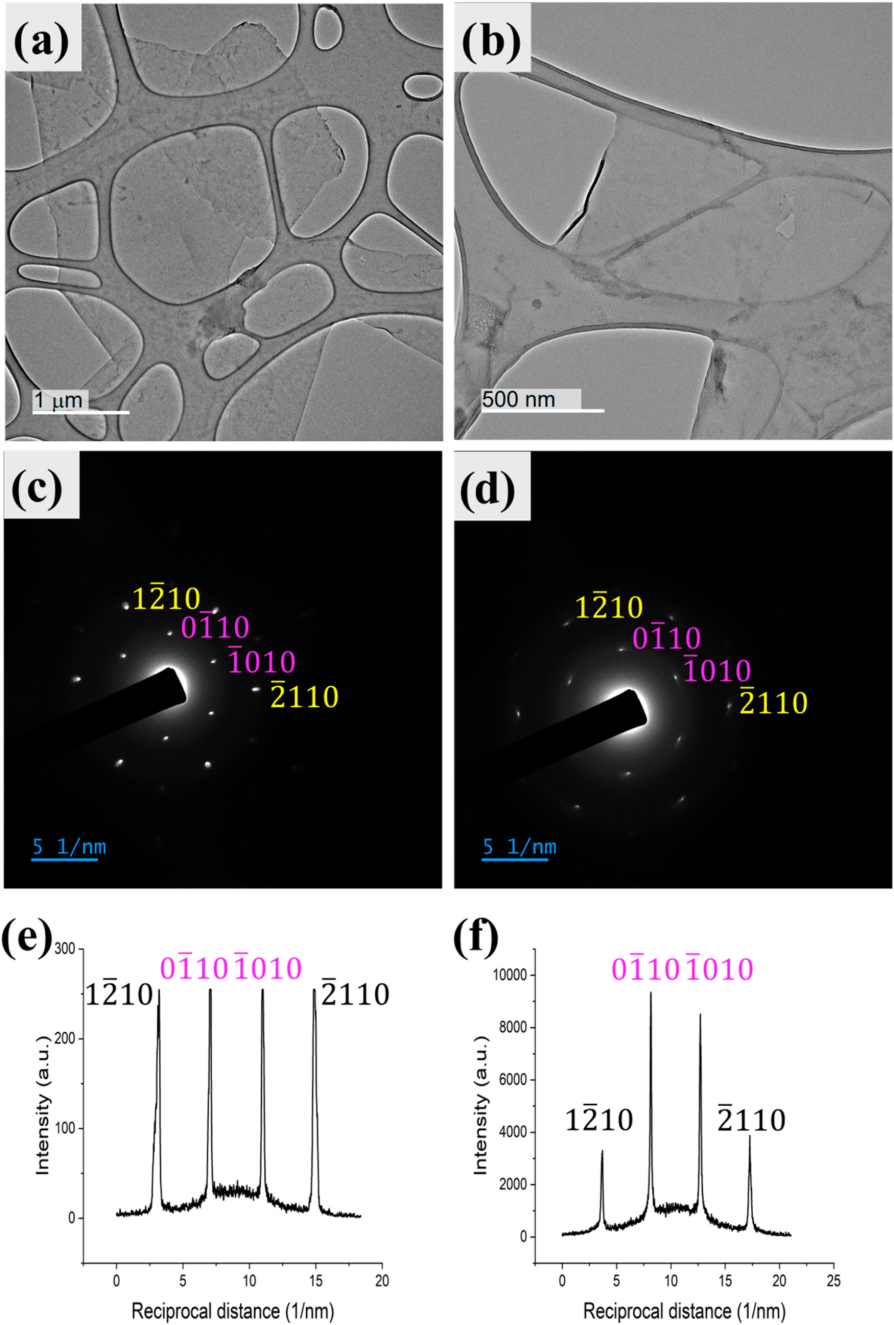
(a, b) TEM image, (c, d) SAED patterns, and (e, f) intensity profile pattern of EEG-N_3_ for bilayer and monolayer flakes, respectively.

TGA analysis can provide additional information on the functional groups introduced on the graphene surface. Figure 6a,b shows that graphite foil is thermally stable up to ∼700 °C. However, EEG and EEG-N_3_ display decomposition peaks within the 150–200 °C range, which is more significant for EEG-N_3_. This agrees with earlier work on the functionalization of graphene in sodium azide with isotopically labeled Na^15^N^14^N_2_ that shows grafted azide groups thermally decompose in the region of 150–200 °C.^31,32^ It is important to note that to mitigate the impact of adsorbed sodium azide, samples were thoroughly washed with deionized water through repeated redispersion and filtration. Figure 6 shows that EEG-N_3_ undergoes 11.6% weight loss, in contrast to EEG’s 4.54% weight loss. Therefore, the significant variation in weight loss cannot be explained by the residual adsorption of NaN_3_.

**Figure 6:**
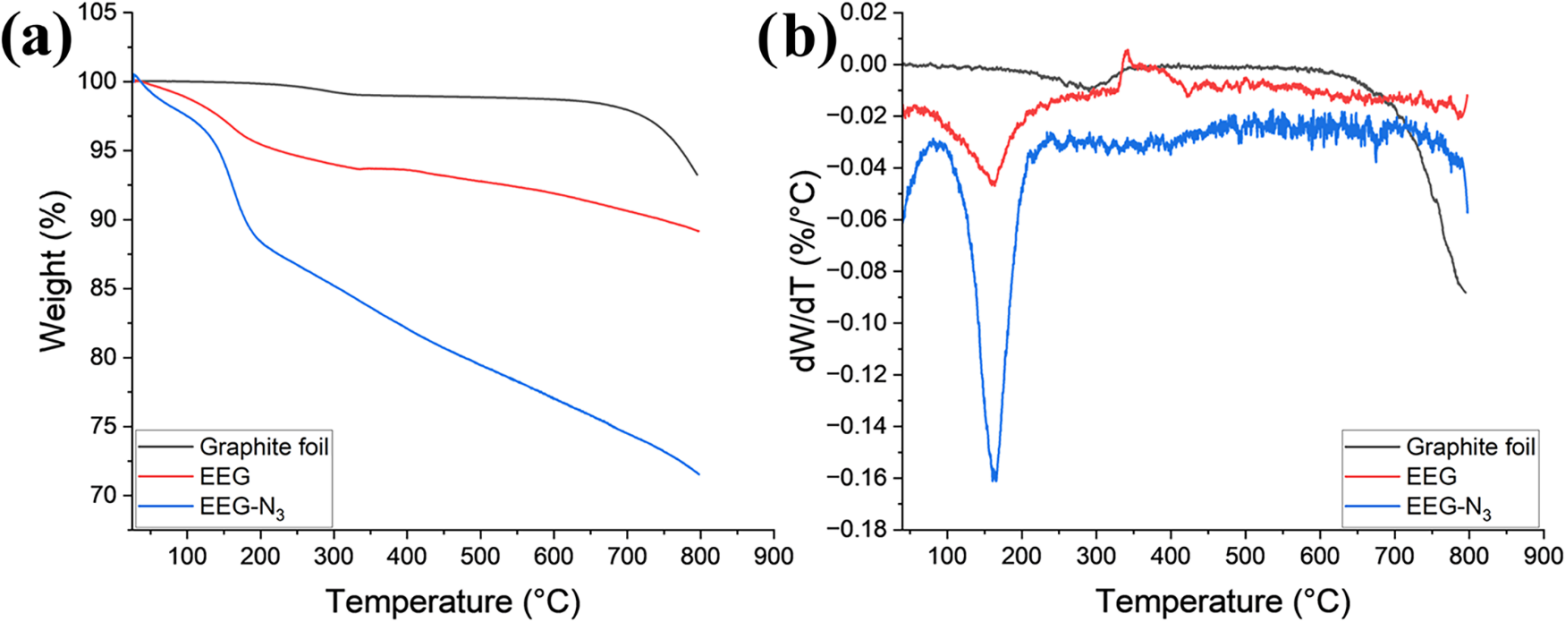
(a) Comparison TGA and (b) first derivative of the weight lost for graphite foil (black), EEG (red), and EEG-N_3_ (blue).

Raman spectroscopy can provide useful information about the structural defects of the graphene flakes.^33^ Graphene exhibits three characteristic peaks, D ∼ 1330 cm^–1^ (associated with sp^3^-hybridization, which is indicative of edges and structural defects), G ∼ 1580 cm^–1^ (related to sp^2^-hybridization), and 2D ∼ 2680 cm^–1^ (used to indicate the number of layers).^33^ The *I*_D/G_ ratio correlates with the density of defects and functional groups and an additional contribution from graphene sheet edges.^34,35^ Figure 7 shows the Raman spectra of graphite foil, EEG, EEG-N_3_, and EEG-TU. The graphite foil shows the expected characteristic 2D peak components at approximately 2681 cm^–1^ (2D_1_) and 2721 cm^–1^ (2D_2_), along with G peaks at 1580 cm^–1^.^36^ However, for the exfoliated samples (EEG and EEG-N_3_), a defect-related D peak appears at ∼1348 cm^–1^ with the D + D′ peak at ∼2940 cm^–1^. The *I*_D_/*I*_G_ and *I*_2D_/*I*_G_ ratios are 1.19 and 0.21 for EEG, 1.35 and 0.20 for EEG-N_3_, and 0.96 and 0.22 for EEG-TU, respectively.

**Figure 7:**
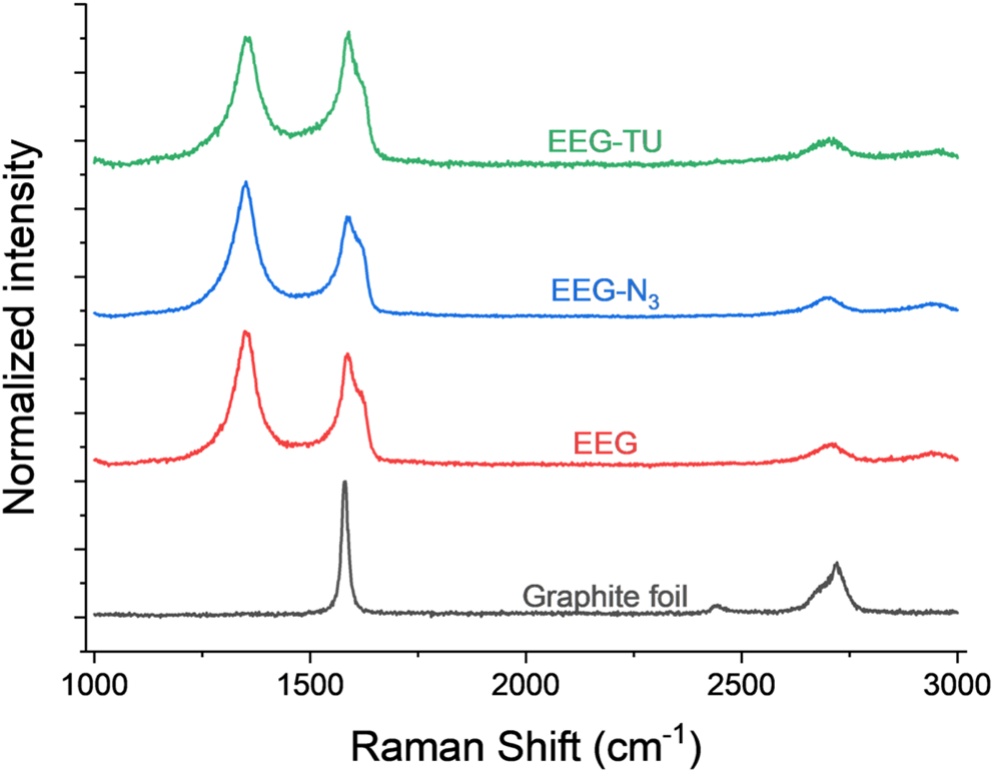
Raman spectra of graphite foil (gray), EEG (red), EEG-N_3_ after azidation (blue), and EEG-TU.

The significant increase in D peak intensity in all the EEG samples, compared to the graphite foil, is attributed to the functionalization of exfoliated graphene with azide groups and some oxygen moieties (as a result of sulfate ion intercalation). However, as the D peak appearance is associated with the disruption of the symmetry of the graphene lattice, it does not provide direct information on the chemical nature of the defects.

X-ray photoelectron spectroscopy (XPS) analysis was used to further identify the chemical groups on the surface of EEG-N_3_. Figure 8a shows the XPS survey scan and Figure 8b,c shows the high-resolution scan of carbon (1s) and nitrogen (1s) regions of EEG-N_3_, respectively. Quantitative analysis shows a N/C atomic ratio of 0.01 and a C/O ratio of 3.52 (after subtracting the oxygen signal from the SiO_2_ substrate, which is lower than that obtained under similar conditions)^8^. Figure 8b shows the deconvoluted XPS spectra of the C 1s peak, indicating the presence of C sp^2^ (284.36 eV), C sp^3^ (284.86 eV), C–O/C-N (286.47 eV), C=O (287.70 eV), and C=C···O (288.50 eV) bonds.^8,37^ From the high-resolution spectra in the N 1s region, four components were fitted. Peaks were assigned to N–C at 398.9 eV,^10,38^ −N=N^+^=N ^–^ at 400.17 eV, −N=N ^+^=N^–^ at 403.44 eV,^39–42^ and N–O 407.05 eV.^43,44^ Therefore demonstrating that the azide group has been successfully introduced to the graphene surface by simultaneous electrochemical and azide functionalization.

**Figure 8:**
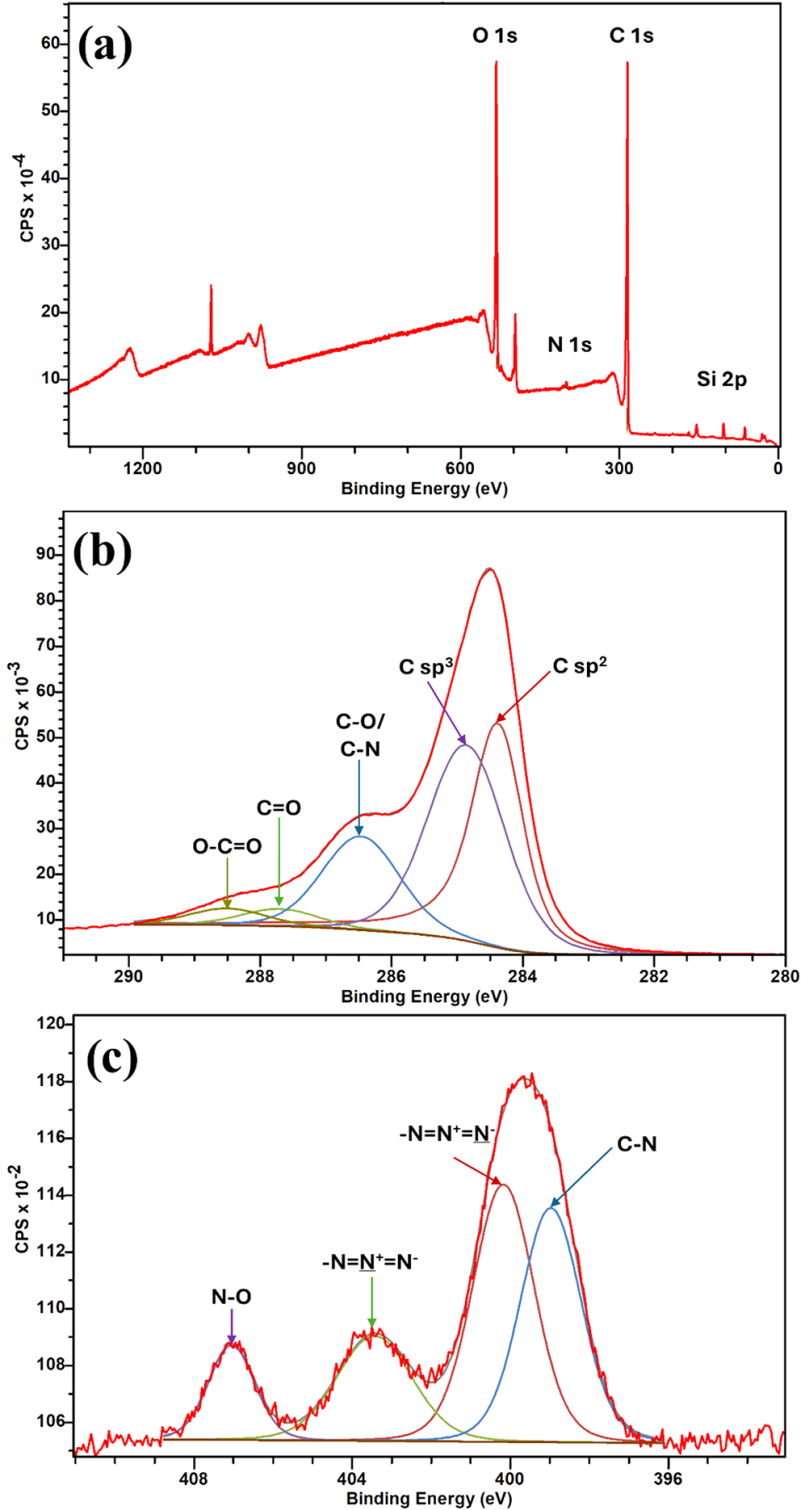
(a) XPS survey scan, (b) high-resolution C 1s region, and (c) high-resolution N 1s region for EEG-N_3_.

The synthesized thiourea selector, (1-(3,5-bis­(trifluoromethyl)­phenyl)-3-(prop-2-yn-1-yl) thiourea), was attached to the azidated graphene surface via CuAAC click chemistry, as detailed in Sections S2 and S3 of the Supporting Information. The XPS survey scan and high-resolution C 1s and N 1s scans of EEG-TU are shown in Figure 9a–c, respectively. The quantitative analysis shows a N/C atomic ratio of 0.048 and a C/O ratio of 3.9 after the oxygen signal is subtracted from the SiO_2_ substrate. As expected, the increase in these ratios is due to the introduction of the thiourea derivative selector moiety. Also, the F 1s peak confirms the bonding of the selector. The C 1s scan shown in Figure 9b was deconvoluted in seven peaks: sp^2^ C (284.45 eV), sp3 C (284.95 eV), C–O/C–N (286.24 eV), C=O (287.26 eV), O=C–O (288.59 eV), C graphitic (291.18 eV), and CF_3_ (292.84 eV).^8,37,45^ Figure 9c shows the spectrum from the N 1s region; the peaks at 398.87 and 400.32 eV correspond to N–C/N–H and N=N, respectively.^43,46^ The absence of the ∼404 eV peak, from the central nitrogen in the azide group, is due to the formation of the triazole ring, confirming the functionalization of the azidated graphene with the selector molecule.^39^

**Figure 9:**
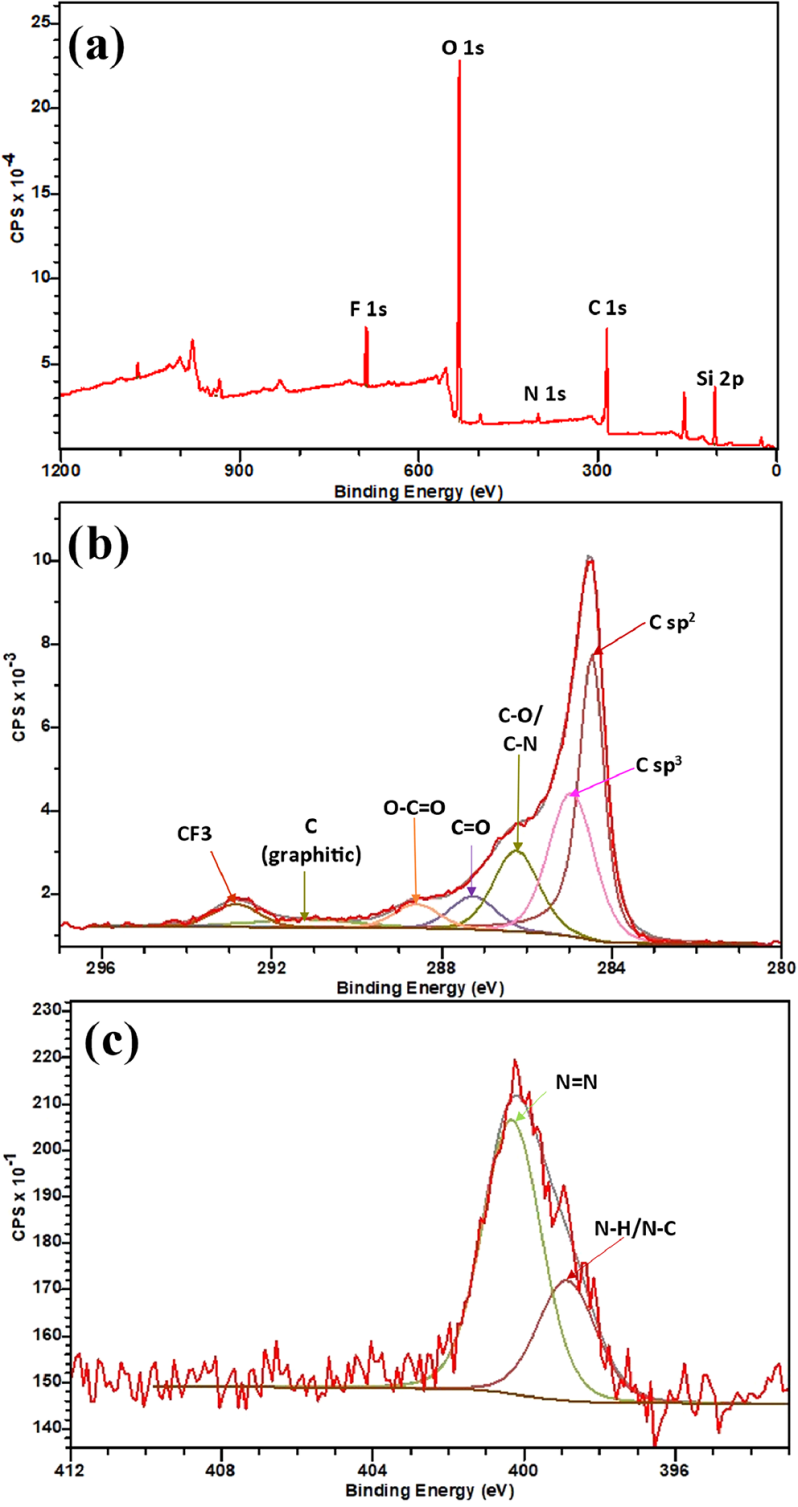
(a) XPS survey scan, (b) high-resolution C 1s region, and (c) high-resolution N 1s region after click chemistry (EEG-TU).

A conductive network is an essential requirement in the development of graphene-based chemiresistors. Hence, the sheet resistance was measured to track the changes in graphene electrical properties during the functionalization steps. As seen in Figure 10, covalent functionalization results in a change to the graphene electrical properties. The disruption of the carbon lattice after introducing azide groups and the thiourea derivative selector increases the sheet resistance compared to graphite foil and EEG.^8,47^ However, even when resistivity is increased, the electrical network is still preserved, making the development of graphene-based chemical resistors still possible.

**Figure 10:**
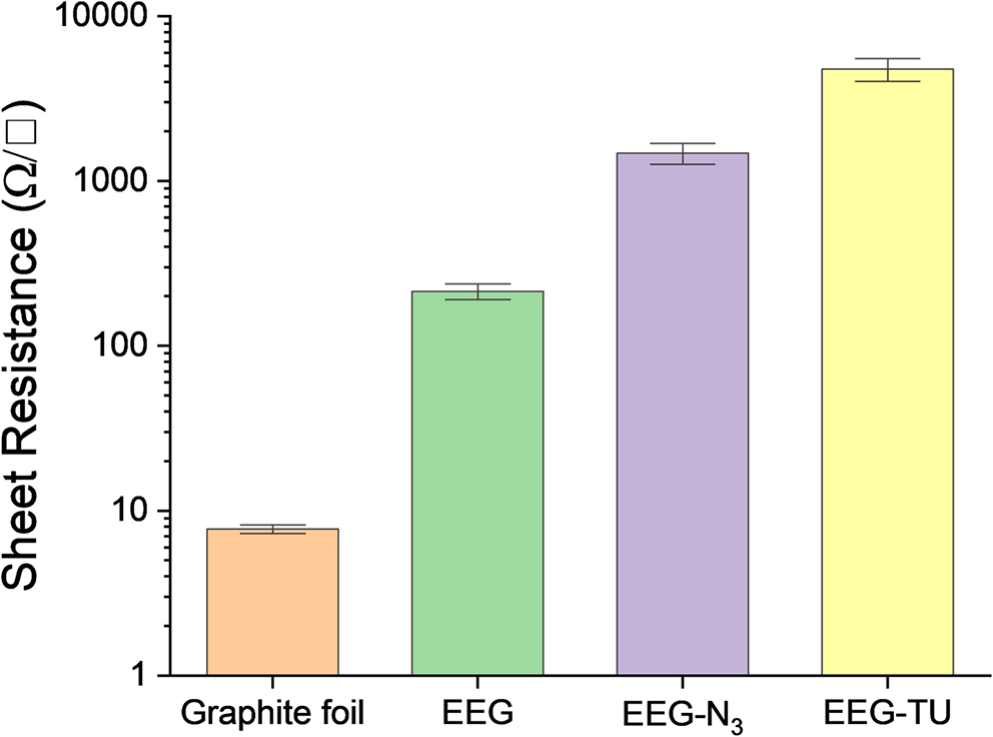
Sheet resistance after each functionalization step; three different positions were measured for each sample.

EEG and EEG-TU sensors were studied and tested in triplicate to determine their responses to cyclohexanone and other volatile organic compounds (VOCs). Figure 11a,b shows the raw data, and Figure 11c shows the sensor response of nonfunctionalized and functionalized sensors after baseline correction. The average response time is 47 and 40 s, respectively. Meanwhile, the average recovery time is approximately 40 s in both cases. The baseline drift can be attributed to the low desorption rates of the gas molecules between adjacent graphene flakes.^48^ The average response of three different devices exposed to 50 ppm cyclohexanone for EEG and EEG-TU was 0.085% and 0.715%, respectively. The average response from three different devices is 8 times higher with the selector group covalently attached to the EEG flakes than with nonfunctionalized graphene.

**Figure 11:**
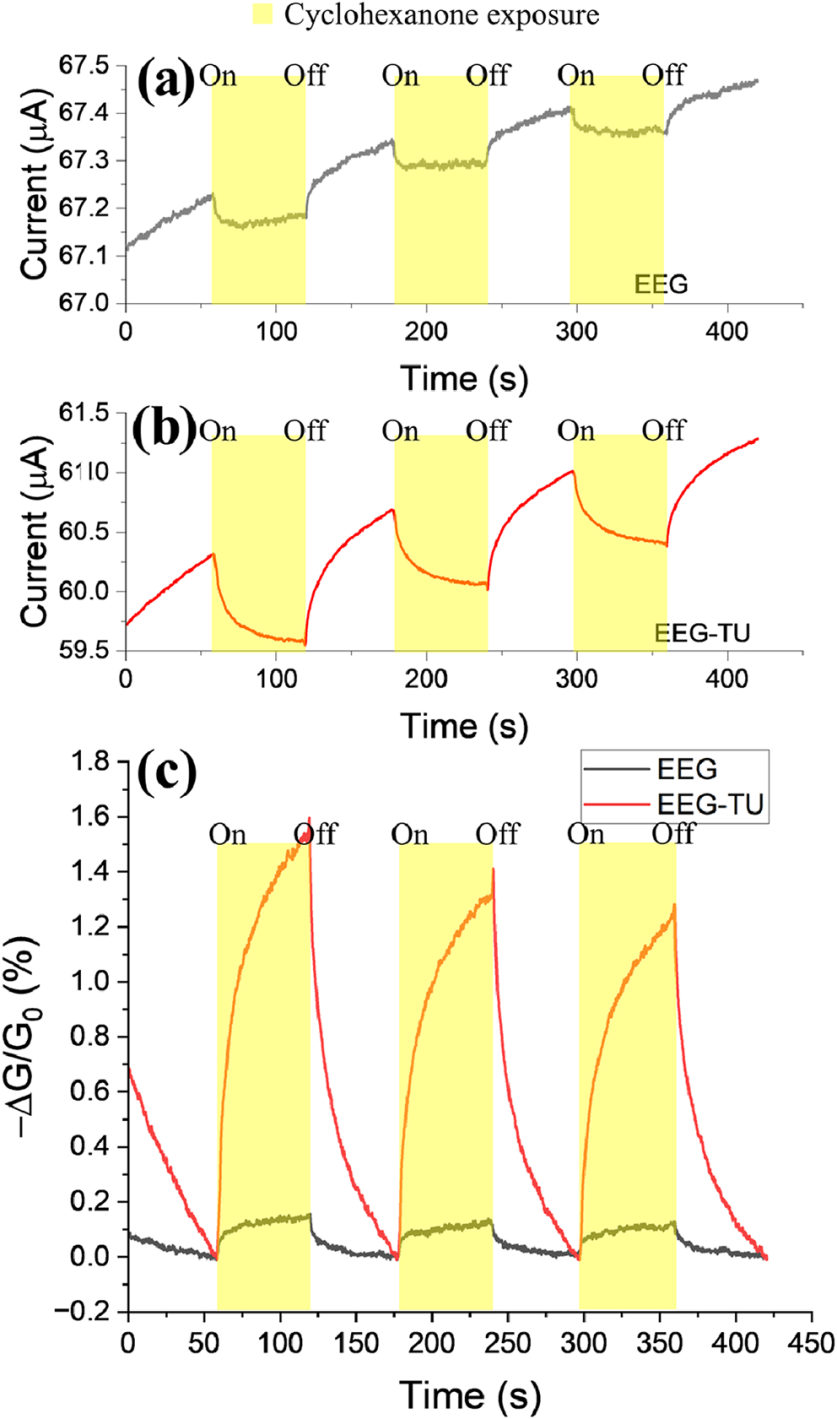
Current change in (a) EEG and (b) EEG-TU sensors. (c) Normalized response after baseline correction, under 50 ppm cyclohexanone exposure for 60 s and followed by flushing with argon for 60 s.

The average response of EEG-based sensors exposed to different concentrations of cyclohexanone vapors, with and without the selector, is plotted in Figure 12. The nonzero Y intercept shown in Figure 12 is caused by the baseline drift and has been observed in other studies on gas sensors.^23^ The baseline drift comes from variations in the environment such as humidity and temperature, voltage fluctuations from the power supply, and the slow rate of adsorption–desorption phenomena.^48,49^ Table 1 shows the summary of the performance parameters for the graphene-based gas sensors where the functionalized sensor (EEG-TU) outperforms the nonfunctionalized sensors (EEG), highlighting the role of the thiourea-based sensing molecule in improving the sensitivity of the device. This is further supported by the estimations of the limit of detection. For EEG-based sensors with and without functionalization, the limits of detection were 4.55 and 27.24 ppm, respectively. Both the limit of detection and response to ∼50 ppm cyclohexanone is comparable to previously reported work on functionalized CNTs with similar or longer exposure times.^23–25^ The slightly reduced sensitivity of the sensors reported here compared to previous research is balanced by a simpler process for introducing the sensing molecule, while still preserving good electrical conductivity for the development of chemiresistive devices.

**Figure 12:**
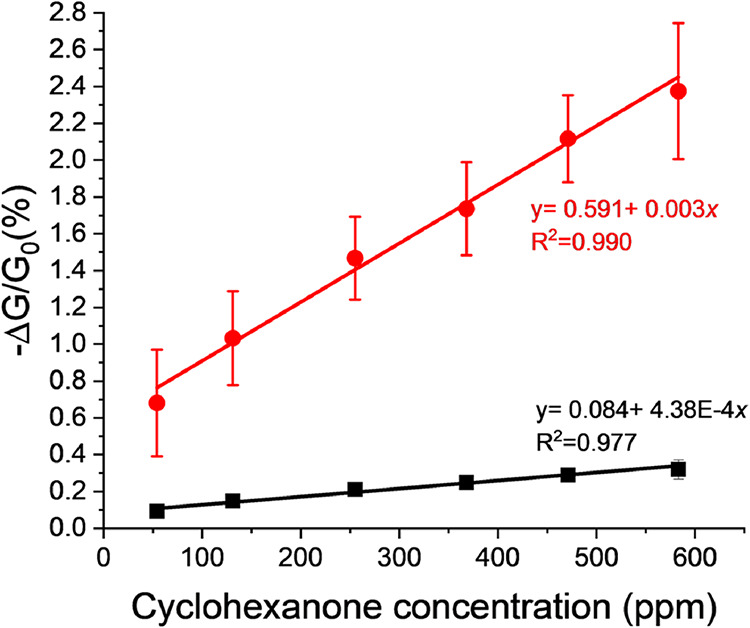
Sensor response for EEG (black line) and EEG-TU devices (red line) exposed to various concentrations of cyclohexanone for 60 s and allowed to recover for 60 s.

**Table 1:** Summary of Graphene-Based Gas Sensor Performance Parameters under Cyclohexanone Exposure

	response at 50 ppm (%)	LOD (ppm)	sensitivity (%/ppm)	response time (s)	recovery time (s)
EEG	0.085	27.24	4.4 × 10^–4^	47	40
EEG-TU	0.715	4.55	3.0 × 10^–3^	40	40

The improvement in sensing response can be linked to the high density of defects in the graphene lattice. Yeo et al. have shown that the sensing responses of defective graphene increase as the number of defects increases.^50^ The decrease in the response time after functionalization reflects the effect of the sensing molecule (TU) on the attraction of the cyclohexanone analyte.^23,25^ The similar recovery times for functionalized and nonfunctionalized devices can be explained bythe strong adsorption on the defect sites, as shown by Zhang et al.^50,51^

Both sensors, EEG and EEG-TU, were tested under exposure to different VOCs (cyclohexanone, acetone, hexane, and ethanol), Figure 13. Their responses were compared by exposing the devices to 50 ppm of each VOC as they have different vapor pressures. The average response is shown in Figure 13, demonstrating that including a selector molecule (TU) improves the selectivity to the analyte. Similarly, functionalized devices can increase the response up to 8 times compared to the nonfunctionalized devices.

**Figure 13:**
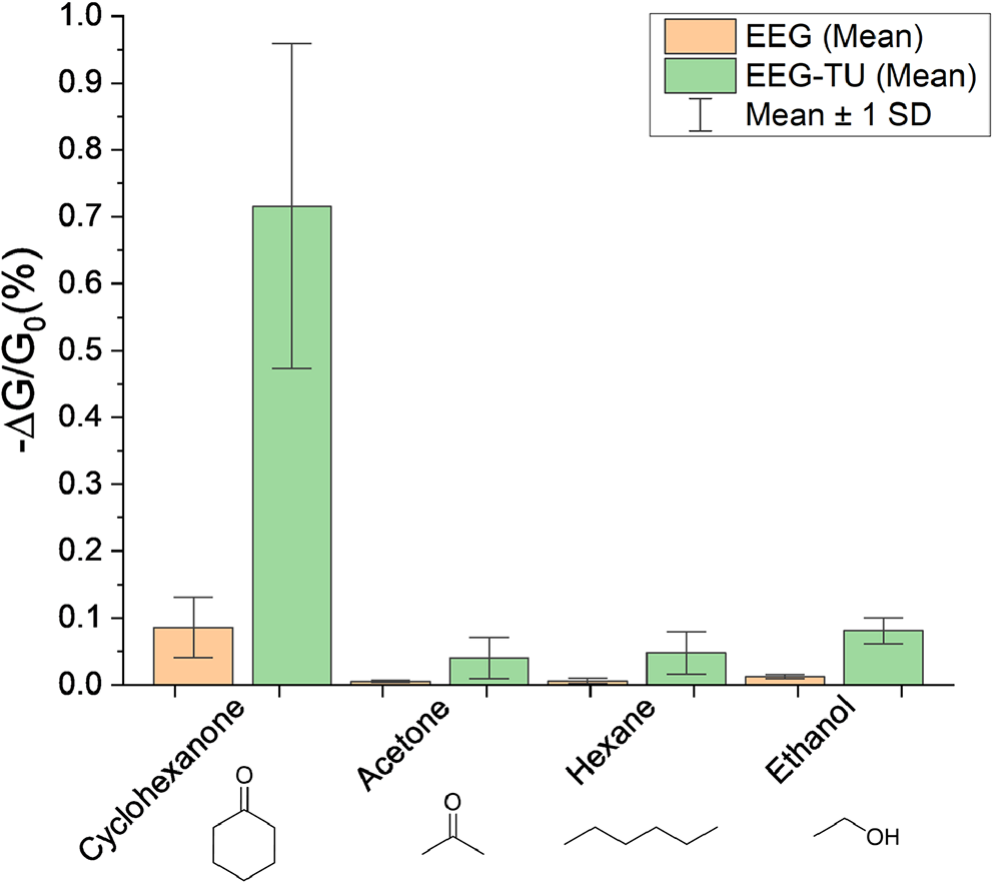
Plot of the normalized average conductive change of EEG and EEG-TU under 50 ppm VOC vapor exposure for 60 s and 60 s recovery times.

Among all of the VOCs tested, the sensor exhibits high sensitivity and selectivity to cyclohexanone when exposed to the same concentrations. The detection of cyclohexanone has been observed in sensors utilizing carbon nanotubes (CNTs).^23–25^ However, the precise mechanism behind their preferential response to cyclohexanone over other molecules has yet to be elucidated. First, we need to consider that networks formed by graphene nanosheets are junction-limited, i.e., the graphene–graphene junction resistance (*R*_J_) is greater than the intrinsic resistance of an individual graphene flake (*R*_Gr_).^52^ Therefore, for the chemical resistor, the resistivity changes observed have a major contribution from *R*_J_. Because the surface of graphene is functionalized with a selector molecule, when the network of functionalized graphene is deposited, the selector molecule can form a bridge where current will flow, inducing a polarization effect on the sensing molecule. This is illustrated in Figure 14.

**Figure 14:**
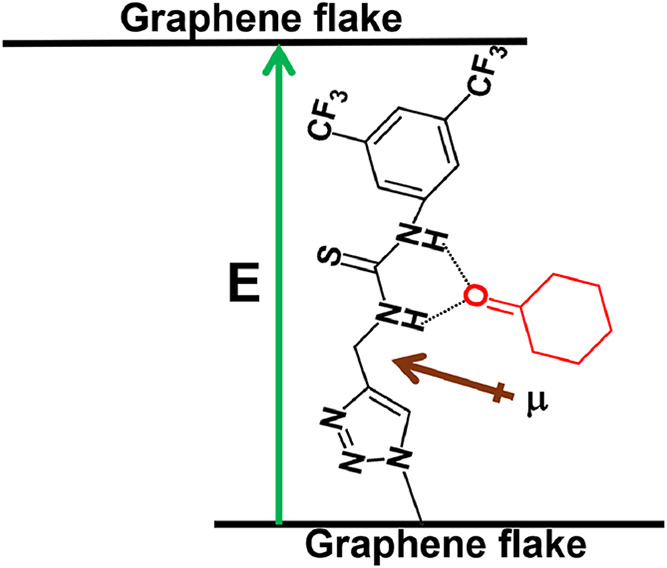
Interaction between the selector and cyclohexanone in covalent functionalized graphene flakes (EEG-TU). The green arrow indicates the electric field (E), and the brown arrow indicates the collective dipole moment (μ).

The examination of the polarizability of the analytes can provide insights into the selectivity toward cyclohexanone. Experiments involving a single molecule as a junction have shown that the resistance (*R*) increases as the polarizability of the tested molecule (α) increases.^53^ Since the polarizability is related to the dipole moment (μ) and the electric field (*E*) as μ = α*E*, a change induced by the dipole–dipole interaction between the sensing molecule and selector should induce a change in the junction resistance. In general, for the dipole induced and a dipole interaction between the selector and the analyte, we can write^54^

2 $$E_{\mathrm{interaction}}\propto \left(\mu_{\mathrm{selector}}^{2}\alpha_{\mathrm{analyte}}+\mu_{\mathrm{analyte}}^{2}\alpha_{\mathrm{selector}}\right)$$

Thus, we should expect a better sensitivity toward those molecules with higher dipolar moments and polarizabilities. In our specific case, it was previously demonstrated that the thiourea-based selector forms a hydrogen-bonded complex with cyclohexanone, enhancing the collective dipolar moment, changing the resistance of the graphene network.^23^ Although this approach must be further explored, it can explain observations from this work and in other studies.^23,25^ While acetone can form hydrogen bonds too, it has a low polarizability, which is consistent with our results presented here and similar previous examples.^23,25^ Moreover, hexane is a nonpolar molecule that cannot form any hydrogen bonding, but it has a high polarizability (11.63 Å^3^);^55^ therefore, it shows a minimal response, comparable to that of acetone.

## 4 Conclusions

This work details the one-step electrochemical synthesis of bulk azidated graphene in an aqueous solution. This differs from previous work, which mainly used GO for azidated graphene, which generates excessive waste and requires an additional reduction step to restore conductivity, which can convert azides to amines, lowering its suitability. The one-pot procedure detailed here is a simple process to prepare azide-functionalized flakes using off-the-shelf graphite foil. Moreover, these functionalized flakes retain a good level of electrical conductivity. The azidated groups were used to introduce a selector molecule through copper­(I)-catalyzed alkyne–azide cycloaddition chemistry, for the selective gas detection of cyclohexanone. The successful click chemistry procedure on simultaneous electrochemically exfoliated and azide-functionalized graphene flakes opens the door to exploring more applications due to their specific functionalization. The devices prepared using EEG or EEG-TU showed a selective response for cyclohexanone when compared to that of other VOCs having a low limit of detection of 4.5 ppm. Here, we provide an explanation for the higher affinity of the selector molecule toward cyclohexanone in terms of the polarizability of the analytes. Although analytes such as acetone and ethanol can form hydrogen bonds, they have polarizabilities much lower than that of cyclohexanone. This agrees with the previous experimental work on CNTs reported by Swager.^23,25^ In conclusion, sensors based on electrochemically exfoliated azidated graphene offer much potential as the exfoliation is simple and azide groups enable ready tailoring of the graphene surface by allowing the introduction of different selector molecules via click chemistry. It is expected that this approach can be expanded and used for the design and development of sensors for other gas analytes such as ammonia, methane, and aromatic species.

## Supplementary Material



## Notes

Detailed experimental information on the exfoliation process; sensing molecule synthesis; click chemistry protocol; description of the gas sensing setup; and computational methods for sensor performance evaluation (PDF)
