# Supplementary material for: Simultaneous Electrochemical Exfoliation of Graphite and Formation of Azide-Functionalized Graphene Flakes and Their Application in Sensing
Source: ACS Omega. 2026 Apr 18;11(17):25874–84. doi: 10.1021/acsomega.6c01334 (PMC13150623; doi:10.1021/acsomega.6c01334)
Supplement: Supplementary file 1 [file ao6c01334_si_001.pdf]

Supplementary information.

Simultaneous electrochemical exfoliation of graphite and formation of azide functionalised graphene flakes and their application in sensing

Selene Muñoz-Vargas[1†], Marcos Fernando Perez-Pucheta[1,2†], and Karl S Coleman[2,*]

[1]Department of Chemistry, Durham University, South Road, Durham DH1 3LE, U.K.

[2]Department of Chemistry, School of Physical Sciences, University of Liverpool, Peach Street, Liverpool L69 7ZE, U.K

*Corresponding authors: karl.coleman@liverpool.ac.uk

S1 Electrochemical exfoliation process.

Materials: Graphite foil (Alfa Aesar, 43078.RF), platinum wire (Good Fellow, PT005130), sodium sulphate (Sigma Aldrich, 238597), sodium azide (Sigma Aldrich, 71289), deionized water, silicon wafers (πPI-KEM, WAFER-SILI-0034).

Graphite foil was used as working electrode (carbon source) and platinum wire as counter electrode. Electrolyte solution of 40 ml of 0.2 M NaN_3 in water and 40 ml of 0.4 M Na_2SO_4 in water were placed in a 100 ml beaker. A constant voltage was applied to the graphite foil (+7 V) until the exfoliation was completed, this was determined when the graphite electrode was detached from the alligator clip. The electrochemical exfoliated graphene with sodium azide groups on its surface (EEG-N_3) was then collected on a nylon filter membrane filter (pore size 0.2 µm) and washed repeatedly with deionized water by vacuum filtration. The resultant EEG was dispersed in water using a bath sonicator (Ultrasonic cleaner, 010S) for 15 min. The dispersion was kept for 24 h to allow for the precipitation of un-exfoliated graphite flakes and/or particles. The top part of the dispersion was used for further characterization and reactions.

[†] Current Address: Department of Chemistry, School of Physical Sciences, University of Liverpool, Peach Street, Liverpool L69 7ZE, U.K.

For electrochemical exfoliated graphene without azide groups on its surface (EEG), 0.4 M Na_2SO_4 in water and +7 V were used, and the process was followed as stated above. The top part of the dispersion was used for further characterization.

The lateral size and thickness of azidated graphene flakes were measured using atomic force microscopy (AFM). 2 ml of the EEG-N_3 dispersion (0.01 mg/ml in 1:6 water:methanol v/v) was added drop-wise onto the water surface in the Langmuir Blodgett trough (611D Nima) using a 1 ml disposable syringe. After spreading, film at the air–water interface was left 20 min to evaporate the solvent. Then the film was compressed at a target pressure of 5 mN/m with a compression rate of 20 cm^2/min. EEG sheets were uniformly deposited on mica by vertically dip coating the substrate with a pull-up rate of 2 mm/min. The samples were left to dry for future Raman and AFM characterizations. For XPS and TEM the sample was prepared by drop cast the dispersion on a SiO_2/Si substrate and holey carbon TEM grid, respectively; they were left to dry overnight. Sheet resistance measurements were conducted on thin films prepared by vacuum filtration on a nylon membrane of aqueous solutions of electrochemical exfoliated graphene flakes, without functionalization (EEG) and with azide groups on its surface (EEG-N_3).

S2 Sensing molecule synthesis.

Materials: Dry Tetrahydrofuran (THF), 3,5-bis(trifluoromethyl)phenyl isothiocyanate (Sima Aldrich, 468517), propargylamine (Fluorochem, 307008), hexane (Fisher Scientific, H/0406/17).

285 μl of propargylamine were added to a solution of 3,5-bis(trifluoromethyl)phenyl isothiocyanate (675 μl) in 10 ml of dry THF, and the mixture was stirred for 5 h. The solvent was removed under reduced pressure. The purification was done by crystallization and vacuum filtration washing the product with hexane. The sensing molecule: 1-(3,5-bis(trifluoromethyl)phenyl)-3-(prop-2-yn-1-yl) thiourea was obtained as a pale yellow powder, it was referred in the text as TU. ^{1}H NMR (599 MHz, DMSO-d_6) δ 10.15 (s, 1H), 8.51 (s, 1H), 8.22 (d, *J* = 1.6 Hz, 2H), 7.73 (s, 1H), 4.31 (s, 2H), 3.19 (t, *J* = 2.5 Hz, 1H), see Figure S1. ^{13}C NMR (151 MHz, DMSO-d_6) δ 180.76, 141.71, 130.48, 130.27, 130.05, 129.83, 125.93, 124.12, 122.40, 122.31, 120.50, 116.51, 80.15, 73.87, 40.06, 39.94, 39.80, 39.66, 39.52, 39.38,

39.24, 39.10, 33.30, see Figure S2. ^{19}F NMR (376 MHz, DMSO-d_6) δ -61.59, see Figure S3. HRMS (ESI) m/z calculated for C12H8N2S1F6 [M+H]+: 327.26, found 327.148 (Figure S4)

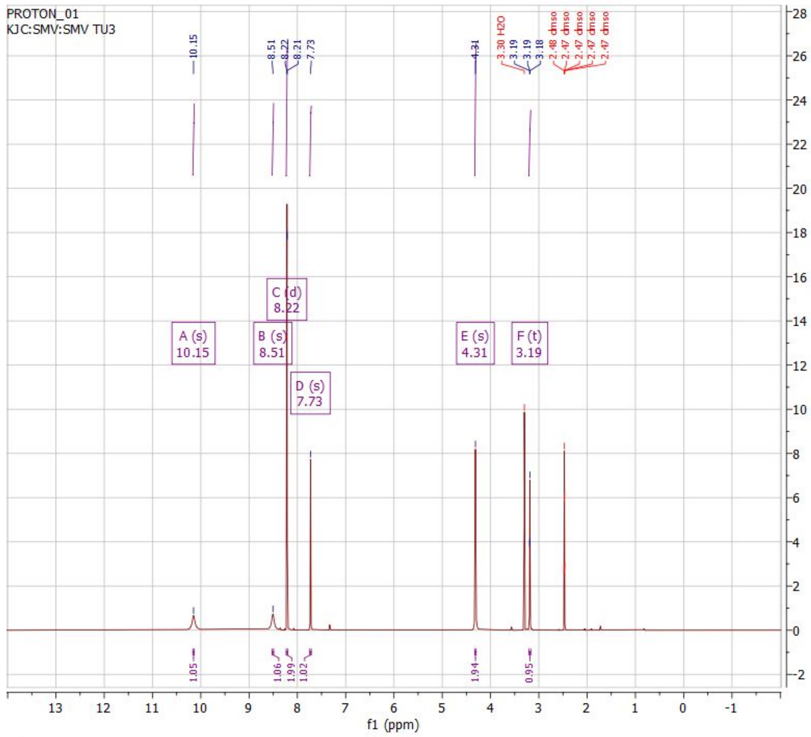

Parameter		Value
1	Data File Name	C:/ Users/ Selene/ Documents/ PROYECTO/ NMR/ Jan30172252/ PROTON_01.fid/ fid
2	Title	PROTON_01
3	Comment	KJC:SMV:SMV TU3
4	Origin	Varian
5	Owner	
6	Site	
7	Instrument	vnmrs
8	Author	
9	Solvent	dmso
10	Temperature	25.0
11	Pulse Sequence	s2pul
12	Experiment	1D
13	Probe	ONE_NMR_MR1101W040
14	Number of Scans	8
15	Receiver Gain	6
16	Relaxation Delay	10.0000
17	Pulse Width	4.2000
18	Presaturation Frequency	
19	Acquisition Time	3.9000
20	Acquisition Date	2023-01-31T12:01:31
21	Modification Date	2023-01-31T11:00:52
22	Class	
23	Spectrometer Frequency	599.17
24	Spectral Width	9615.4
25	Lowest Frequency	-1212.9
26	Nucleus	1H
27	Acquired Size	37500
28	Spectral Size	65536
29	Digital Resolution	0.15

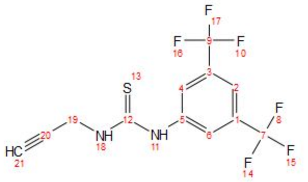

^{1}H NMR (599 MHz, dmso) δ 10.15 (s, 1H), 8.51 (s, 1H), 8.22 (d, J = 1.6 Hz, 2H), 7.73 (s, 1H), 4.31 (s, 2H), 3.19 (t, J = 2.5 Hz, 1H).

Figure S1 ^{1}H NMR Spectrum of TU.

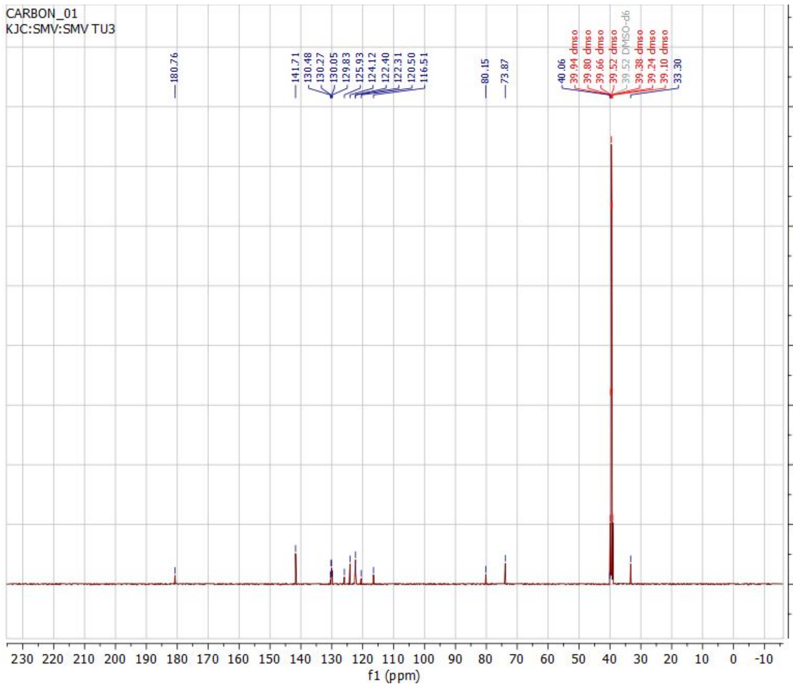

Parameter		Value
1	Data File Name	C:/ Users/ Selene/ Documents/ PROYECTO/ NMR/ Jan30172252/ CARBON_01.fid/ fid
2	Title	CARBON_01
3	Comment	KJC:SMV:SMV TU3
4	Origin	Varian
5	Owner	
6	Site	
7	Instrument	vnmrs
8	Author	
9	Solvent	dmso
10	Temperature	25.0
11	Pulse Sequence	s2pul
12	Experiment	1D
13	Probe	ONE_NMR_MR1101W040
14	Number of Scans	1292
15	Receiver Gain	60
16	Relaxation Delay	0.7000
17	Pulse Width	4.6125
18	Presaturation Frequency	
19	Acquisition Time	3.4603
20	Acquisition Date	2023-01-31T13:41:53
21	Modification Date	2023-01-31T14:08:57
22	Class	
23	Spectrometer Frequency	150.68
24	Spectral Width	37878.8
25	Lowest Frequency	-2432.4
26	Nucleus	13C
27	Acquired Size	131072
28	Spectral Size	262144
29	Digital Resolution	0.14

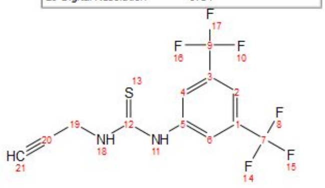

^{13}C NMR (151 MHz, dmso) δ 180.76, 141.71, 130.48, 130.27, 130.05, 129.83, 125.93, 124.12, 122.40, 122.31, 120.50, 116.51, 80.15, 73.87, 40.06, 39.94, 39.80, 39.66, 39.52, 39.38, 39.24, 39.10, 33.30.

Figure S2 ^{13}C NMR Spectrum of TU.

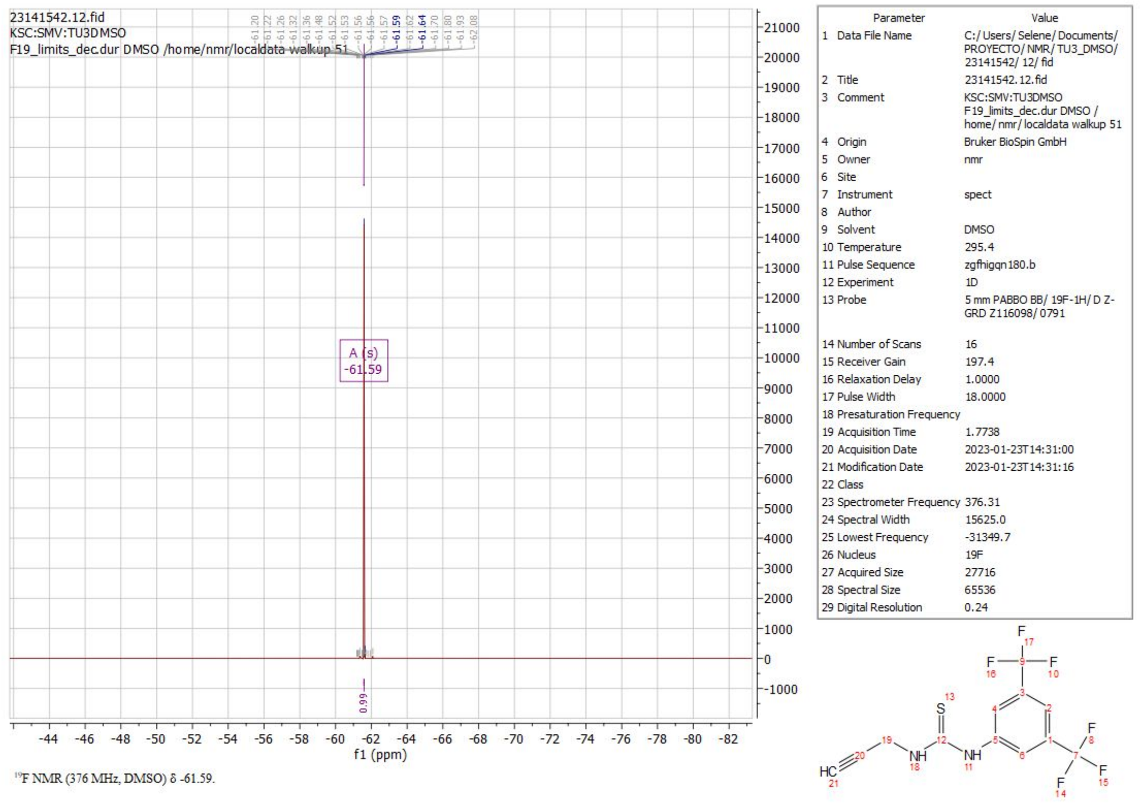

^{19}F NMR (376 MHz, DMSO) δ -61.59.

Figure S3 ^{19}F NMR Spectrum of TU.

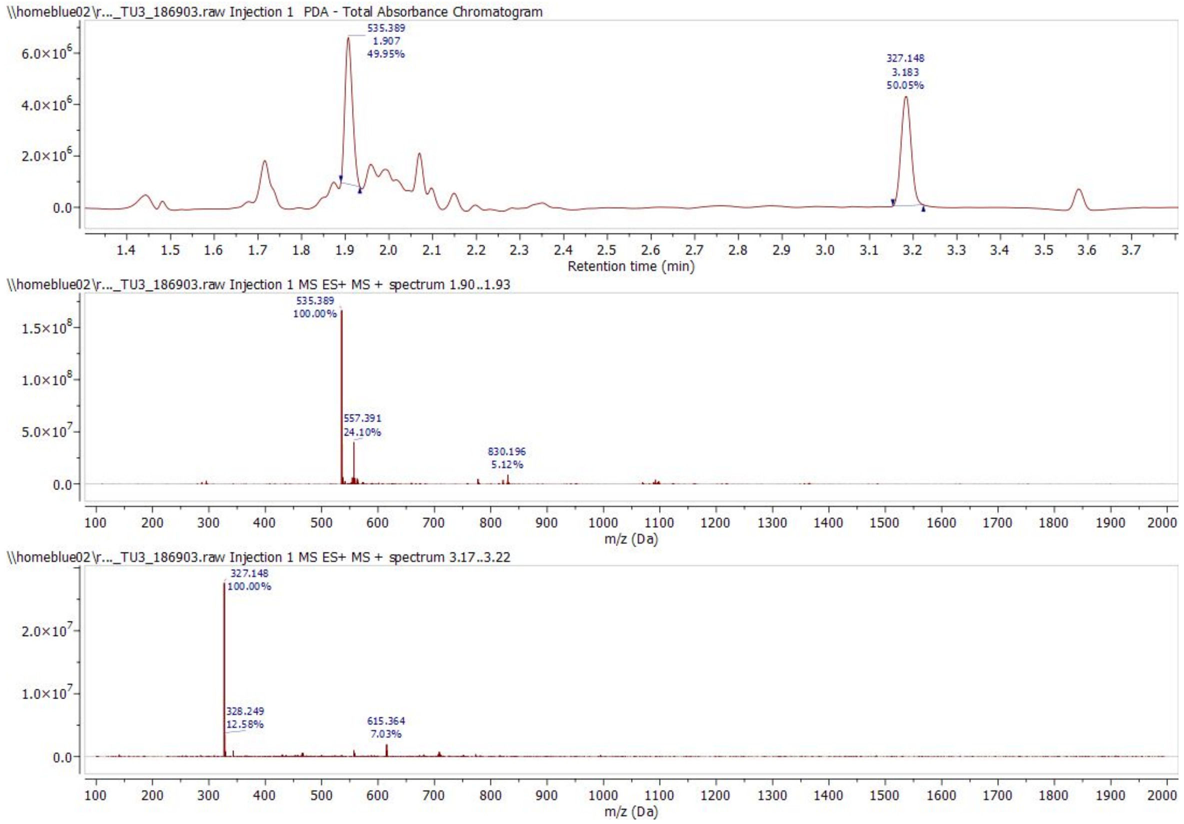

Figure S4 Mass spectroscopy results.

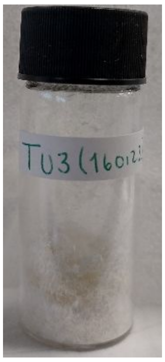

Table S1 Elemental analysis results.

Measured			Expected			Difference		
% C	% H	% N	% C	% H	% N	% C	% H	% N
44.09	2.41	8.55	44.18	2.47	8.58	-0.09	-0.06	-0.03

S3 Click chemistry protocol.

Materials: Electrochemical exfoliated azidated graphene (EEG-N_3), 1-(3,5-bis(trifluoromethyl)phenyl)-3-(prop-2-yn-1-yl) thiourea (TU), methanol (Fisher Scientific, M/4000/17), copper sulphate pentahydrate (Fluorochem, 044725), ascorbic acid (Fluorochem, 093967), and deionized water.

EEG-N_3 were functionalized with the selector as follows: first 47 ml of 1 mM TU solution in methanol was placed in a flask, then 1.3 ml of an aqueous solution of 5 mM $CuSO_4 \cdot 5H_2O$ and 4.4 ml of an aqueous solution of 10 mM ascorbic acid were added to it. This reaction solution was added to a solution of azidated graphene flakes (EEG-N_3) in 35 ml (1:1) water:methanol. The sample was left for reaction to react for one hour.

The washing process involved centrifugation at 10000 rpm for 10 minutes, resulting in the recovery of graphene at the bottom and subsequent removal of the supernatant. The sample was dispersed again in a water:methanol (1:1) solution, and this procedure was repeated twice more. The sample was dispersed in 40 ml water:methanol 1:1 (it is referred as EEG-TU) for further use in sensors and characterizations. For XPS the sample was prepared by drop cast the dispersion on a SiO_2/Si substrate. Sheet resistance measurements were conducted on thin films prepares by vacuum filtration on nylon membranes of EEG-TU solution.

S4 Detailed explanation of the setup used for gas sensing.

This system consists of a vapour-generating bubbler, a carrier gas, and a detection chamber, as illustrated in Figure S5. When the gas carrier passes through the bubbler with the analyte

solution, it saturates the atmosphere. To calculate the saturated vapour pressure of the analyte in the gas chamber, $\boldsymbol{P}_s$, Antoine's equation was used:[1]

$$\log_{10}(P_s) = A - \frac{B}{T + C} \qquad \text{Eq. S.1}$$

where ***A***, ***B***, and ***C*** are unitless constants known as Antoine's constants and ***T*** is the temperature in Kelvin or °C for $\boldsymbol{P}_s$ in bar and mm Hg, respectively. The constants for each of the VOCs tested are summarised in Table S2. The VOC concentration in parts per million is determined using Eq. S.2 .

$$C_{ppm} = \left(\frac{P_s}{P} \times \frac{F_{VOC}}{F_{VOC} + F}\right) \times 10^6 \qquad \text{Eq. S.2}$$

where $\boldsymbol{P_s}$ is the saturated partial pressure in mm Hg, ***P*** is the chamber pressure (760 mm Hg), $\boldsymbol{F_{VOC}}$ is the flow in sccm of the VOC, and ***F*** is the carrier gas (Argon) flow in sccm.

Table S2 Antoine's parameters for each VOC (T in °C and P_s in mm Hg, except for cyclohexanone where T is in K and P_s in bar).

VOC	A	B	C	Reference
Cyclohexanone	4.10	1495.51	-63.60	[2]
Acetone	7.13	1219.97	230.65	[3]
Hexane	7.01	1246.33	232.99	
Ethanol	8.20	1642.89	230.30	

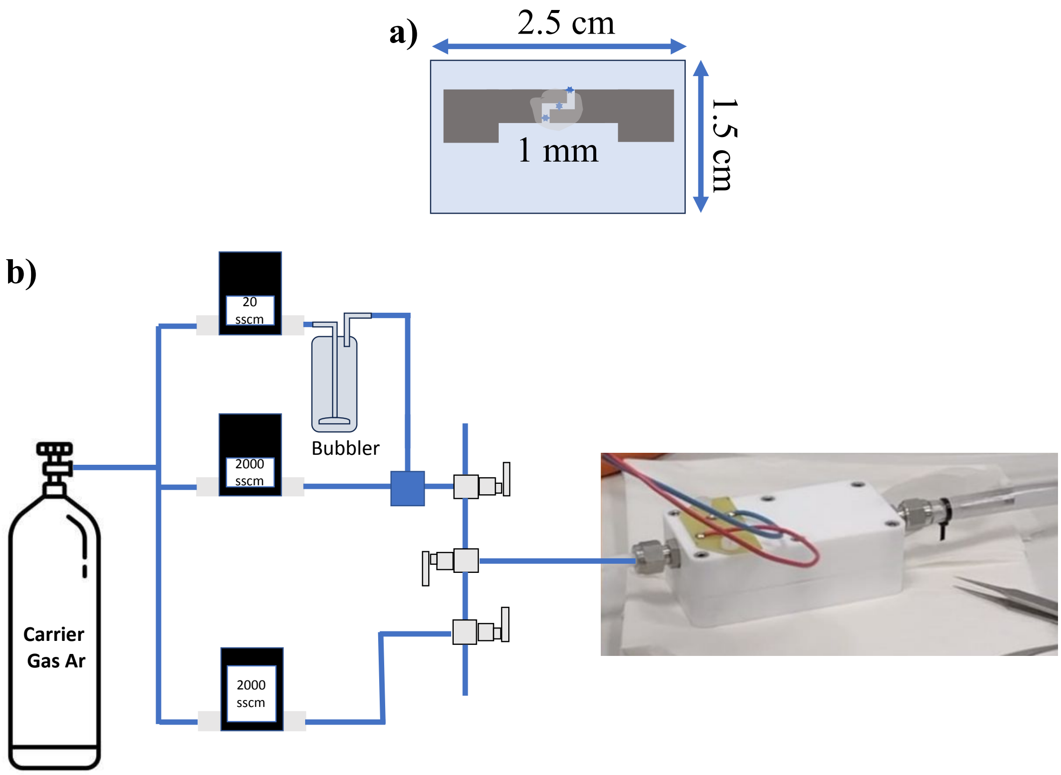

Figure S5 a) Schematic representation of EEG-based sensors where the light grey stain in the middle represents the drop casted EEG flakes, and the dark grey represents the AuPd deposited electrodes that leaves 1 mm channel width (small blue arrows behind the light grey shadow). b) Experimental set up for gas sensing, three mass flow controllers (two for dilution and one for flushing), three three-way valves and the PTFE chamber.

S5 Calculations of sensor parameters performance.

The gas sensor performance can be expressed in terms of the following parameters: sensor response, sensitivity, selectivity, stability, limit of detection (LOD), linearity, response time, and recovery time. Sensor response is defined as the ratio of the variation in the resistance, conductance or current of the system with and without the analyte (gas molecules) in terms of the initial reference value. A large value indicates that material is a good sensor.[4] Here, the normalised response of the sensors was calculated using Eq. S.3.

$$-\frac{\Delta G}{G_0}(\%) = -\frac{I - I_0}{I_0} \times 100 \qquad \text{Eq. S.3}$$

where I_0 is the initial current before exposure to the analyte, and I is the measured current over time. The sensitivity is determined from the slope of the calibration curve between the

sensor response and gas concentration, so it is the rate of change of sensor response per unit change in gas concentration. Another important parameter is the selectivity which is the ability of a sensor material to distinguish between the analyte and other gases. As many of sensors are sensitive to many gases under similar operating conditions, the selectivity compares the signal of the sensor at the same concentration of the corresponding interfering gas.[4]

In this work, the response time was considered as the time interval over which the sensor response is 90% of the final value when it is exposed to a specific gas concentration. On the other hand, the recovery time was considered as the interval over which the sensor response reduces to 10% of its final value (after exposure to the target gas) when flushing with argon gas.[5]

Limit of detection (LOD) is the lowest concentration of the analyte which can be detected by the sensor. The theoretical LOD for cyclohexanone was calculated similarly to Li *et al.*[6] In brief, the noise for each sensor was calculated using the deviation in the response (-$\Delta G/G_0$, %) from the baseline before exposing the sensor to the analyte. Twenty-one points were taken before exposure to the cyclohexanone, and data were plotted and fitted to a 5th-order polynomial. Then, the residual sum of squares was calculated according to Eq. S.4, where $\boldsymbol{y_i}$ is the measured data point, and $\boldsymbol{y}$ is the corresponding value from 5-th order polynomial fit. The root-mean-square noise ($\boldsymbol{rms_{noise}}$) is computed with Eq. S.5, being $\boldsymbol{N}$, the number of data points used for curve fitting. It is considered a valid signal when the signal-to-noise ratio is equal to 3, and the LOD can be determined according to Eq. S.6, where the slope corresponds to the linear regression fit from the calibration curve obtained at different concentrations of cyclohexanone.

$$V_{\chi^2} = (y_i - y)^2 \qquad \text{Eq. S.4}$$

$$rms_{noise} = \sqrt{\frac{V_{\chi^2}}{N}} \qquad \text{Eq. S.5}$$

$$LOD = 3\,\frac{rms_{noise}}{slope} \qquad \text{Eq. S.6}$$

References.

(1) Dean, J. A. *Lange's Handbook of Chemistry*; 1999.

(2) Meyer, E. F.; Hotz, R. D. High-Precision Vapor-Pressure Data for Eight Organic Compounds. *J. Chem. Eng. Data* **1973**, *18* (4), 359–362. https://doi.org/10.1021/je60059a008.

(3) GmbH, D. *Saturated Vapor Pressure Calculation by Antoine Equation*. http://ddbonline.ddbst.com/AntoineCalculation/AntoineCalculationCGI.exe.

(4) Sabu, T.; Nirav, J.; Vijay K, T. *Functional Nanomaterials*; Thomas, S., Joshi, N., Tomer, V. K., Eds.; Materials Horizons: From Nature to Nanomaterials; Springer Singapore: Singapore, 2020. https://doi.org/10.1007/978-981-15-4810-9.

(5) Sidhu, S. S.; Redouane, Z.; Preetkanwal, B. S.; Morteza, Y. *Materials Horizons : From Nature to Nanomaterials Futuristic Composites*; 2018.

(6) Li, J.; Lu, Y.; Ye, Q.; Cinke, M.; Han, J.; Meyyappan, M. Carbon Nanotube Sensors for Gas and Organic Vapor Detection. *Nano Lett.* **2003**, *3* (7), 929–933. https://doi.org/10.1021/nl034220x.
